# Improving the use of research evidence in guideline development: 8. Synthesis and presentation of evidence

**DOI:** 10.1186/1478-4505-4-20

**Published:** 2006-12-05

**Authors:** Andrew D Oxman, Holger J Schünemann, Atle Fretheim

**Affiliations:** 1 Norwegian Knowledge Centre for the Health Services, P.O. Box 7004, St. Olavs plass, N-0130 Oslo, Norway; 2 INFORMA, S.C. Epidemiologia, Istitituto Regina Elena, Via Elio Chianesi 53, 00144 Rome, Italy

## Abstract

**Background:**

The World Health Organization (WHO), like many other organisations around the world, has recognised the need to use more rigorous processes to ensure that health care recommendations are informed by the best available research evidence. This is the eighth of a series of 16 reviews that have been prepared as background for advice from the WHO Advisory Committee on Health Research to WHO on how to achieve this.

**Objectives:**

We reviewed the literature on the synthesis and presentation of research evidence, focusing on four key questions.

**Methods:**

We searched PubMed and three databases of methodological studies for existing systematic reviews and relevant methodological research. We did not conduct systematic reviews ourselves. Our conclusions are based on the available evidence, consideration of what WHO and other organisations are doing and logical arguments.

**Key questions and answers:**

We found two reviews of instruments for critically appraising systematic reviews, several studies of the importance of using extensive searches for reviews and determining when it is important to update reviews, and consensus statements about the reporting of reviews that informed our answers to the following questions.

How should existing systematic reviews be critically appraised?

• Because preparing systematic reviews can take over a year and require capacity and resources, existing reviews should be used when possible and updated, if needed.

• Standard criteria, such as A MeaSurement Tool to Assess Reviews (AMSTAR), should be used to critically appraise existing systematic reviews, together with an assessment of the relevance of the review to the questions being asked.

When and how should WHO undertake or commission new reviews?

• Consideration should be given to undertaking or commissioning a new review whenever a relevant, up-to-date review of good quality is not available.

• When time or resources are limited it may be necessary to undertake rapid assessments. The methods that are used to do these assessments should be reported, including important limitations and uncertainties and explicit consideration of the need and urgency of undertaking a full systematic review.

• Because WHO has limited capacity for undertaking systematic reviews, reviews will often need to be commissioned when a new review is needed. Consideration should be given to establishing collaborating centres to undertake or support this work, similar to what some national organisations have done.

How should the findings of systematic reviews be summarised and presented to committees responsible for making recommendations?

• Concise summaries (evidence tables) of the best available evidence for each important outcome, including benefits, harms and costs, should be presented to the groups responsible for making recommendations. These should include an assessment of the quality of the evidence and a summary of the findings for each outcome.

• The full systematic reviews, on which the summaries are based, should also be available to both those making recommendations and users of the recommendations.

What additional information is needed to inform recommendations and how should this information be synthesised with information about effects and presented to committees?

• Additional information that is needed to inform recommendations includes factors that might modify the expected effects, need (prevalence, baseline risk or status), values (the relative importance of key outcomes), costs and the availability of resources.

• Any assumptions that are made about values or other factors that may vary from setting to setting should be made explicit.

• For global guidelines that are intended to inform decisions in different settings, consideration should be given to using a template to assist the synthesis of information specific to a setting with the global evidence of the effects of the relevant interventions.

## Background

The World Health Organization (WHO), like many other organisations around the world, has recognised the need to use more rigorous processes to ensure that health care recommendations are informed by the best available research evidence. This is the eighth of a series of 16 reviews that have been prepared as background for advice from the WHO Advisory Committee on Health Research to WHO on how to achieve this.

A summary of the best available research evidence is essential, though not sufficient to inform recommendations. To reduce the risk of bias and errors that occur by chance, and to facilitate critical appraisal of syntheses of evidence, reviews should be systematic and should explicitly report the methods that were used [1]. However, systematic reviews require resources, take time, and may not always be warranted or possible. Moreover, unnecessary duplication of systematic reviews should be avoided, given the large unmet need for systematic reviews of a wide range of questions and the need to keep reviews up-to-date [2,3].

The first step in considering the needs for systematic reviews to inform recommendations is to critically appraise existing reviews to determine if they provide an adequate summary of the relevant evidence that is needed, particularly evidence of the effects of the different options (interventions) that are being considered. If they do not, consideration must then be given to whether a new review should be undertaken and how best to obtain a new review. Once an adequate summary of the evidence is available, consideration must be given to how best to present that information to the group of people who will consider that evidence, together with other evidence and judgements, to develop recommendations. In addition, consideration needs to be given to the additional information that is needed and how that should be summarised and presented.

In this paper we address the following questions:

• How should existing systematic reviews be critically appraised and used?

• When and how should WHO undertake or commission new reviews?

• How should the findings of systematic reviews be summarised and presented to committees responsible for making recommendations?

• What additional information is needed to inform recommendations and how should this information be synthesised with information about effects and presented to committees?

Related questions on priority setting for guidelines or recommendations and reporting of guidelines are addressed in other papers in this series [4,5].

## What WHO is doing now

Of 62 WHO documents that were indexed as guidelines in 2005, only two reported a systematic review and less than 40% included references [6]. Although it is possible that systematic reviews are being used and this is not being reported, this is unlikely. With some notable exceptions, for the most part recommendations are currently being made without adequate use of existing systematic reviews and systematic reviews are rarely being undertaken or commissioned by WHO committees that make recommendations. However, the situation may be somewhat better than what is reported in published guidelines. When asked about the use of evidence of effects specifically in an interview study [7], many departments reported using background documents. These were reported to have been prepared in a variety of ways, including as unpublished working papers, documents similar to those used by the Scottish Intercollegiate Guidelines Network (SIGN), and documents prepared by the participating experts. Only two departments reported using systematic reviews specifically, while several others reported using systematic reviews along with a range of other documents. Others reported leaving the use of evidence up to the experts, a lack of documentation, evidence of effects not being relevant for some recommendations, and using a mixture of "epidemiological data, trial data, opinions based on logical reasoning (common sense) and clinical experience."

No departments reported using concise summaries of findings or "balance sheets" for the most important outcomes (benefits, harms and costs) for the options that were considered. WHO groups that develop recommendations are, for the most part, composed of experts in a particular content area and not supported by experts in particular methodological areas (e.g. systematic reviews) or by staff with particular technical skills (e.g. information retrieval). Relatively little attention appears to have been given to how best to help member states adapt global recommendations, taking account of local needs, values, resources and conditions.

## What other organisations are doing

In contrast, in a survey of 101 organisations that produce clinical practice guidelines 95% of the 58 respondents reported that they provide guideline panels with systematic reviews [8]. In another survey of 18 prominent organisations that develop clinical practice guidelines, all but one reported using systematic reviews [9].

The UK National Centre for Health and Clinical Excellence (NICE), for example routinely undertakes systematic reviews to inform its guideline panels [10]. NICE has seven professionally led National Collaborating Centres to manage the development of clinical guidelines [11]. Each Centre has a range of skills and abilities, including systematic reviewing. The Centres are responsible for identifying the best and most relevant evidence available. They write the first consultation draft of a guideline over a period of 12 to 18 months. NICE reviews are available in the full version of its guidelines.

Other organisations that produce guidelines sometimes use existing systematic reviews, sometimes prepare their own systematic reviews, and sometimes commission reviews. The U.S. Preventive Services Task Force, for example, commissions systematic reviews from Evidence-based Practice Centers (EPCs) for updates of its guidelines [12]. The Agency for Healthcare Research and Quality (AHRQ) has contracts with 13 EPCs from which it commissions systematic reviews. AHRQ does not produce guidelines, but stakeholder organisations that request the reviews may produce guidelines. Other health technology assessment (HTA) agencies, which may or may not produce guidelines, have staff that undertake reviews, convene expert groups that undertake reviews together with support from staff, or commission systematic reviews [13].

Systematic reviews of the effects of interventions are a major focus for most organisations that develop guidelines. Because most organisations develop recommendations for a specific country or setting, they are able to take into account additional information relevant to the specific context for which the recommendations are intended, including factors that might affect the applicability of the evidence in specific settings, need (prevalence, baseline risk or status), values, costs and the availability of resources.

## Methods

The methods used to prepare this review are described in the introduction to this series [14]. Briefly, the key questions addressed in this paper were vetted amongst the authors and the ACHR Subcommittee on the Use of Research Evidence (SURE). We did not conduct a full systematic review. We searched PubMed and three databases of methodological studies (the Cochrane Methodology Register [15], the US National Guideline Clearinghouse [16], and the Guidelines International Network [17]) for existing systematic reviews and relevant methodological research that address these questions. The answers to the questions are our conclusions based on the available evidence, consideration of what WHO and other organisations are doing, and logical arguments.

For this review we knew of two previous systematic reviews of instruments for critically appraising systematic reviews through personal contacts [18,19], and studies of how to present the results of systematic reviews to policy makers [20], the general public [21], and users of Cochrane reviews [22]. We used these studies and their reference lists to identify related articles in PubMed. We searched the Cochrane Methodology Register using the key word 'Presentation of reviews: General' and we checked the reference lists of the reports that we retrieved. We searched for literature on priority setting for guidelines and health technology assessments for another report [4]. In addition, we searched broadly for literature on commissioning systematic reviews in PubMed (commissioning systematic reviews) and using Google ("commissioning systematic reviews" and "updating systematic reviews") and in the Cochrane Methodology Register using the terms 'commissioning' and 'updating systematic reviews'. The searches were conducted in March 2006.

## Findings

### How should existing systematic reviews be critically appraised?

The first of two reviews of different instruments for critically appraising systematic reviews found 20 systems concerned with the appraisal of systematic reviews or meta-analyses, including one scale, 10 checklists, and nine guidance documents [18]. The authors identified seven key domains that they considered important to appraise: study question, search strategy, inclusion and exclusion criteria, data abstraction, study quality, data synthesis and analysis, and funding or sponsorship. One checklist fully addressed all seven domains [23]. A second checklist also addressed all seven domains but merited only a "Partial" score for study question and study quality [24]. Two additional checklists and the one scale addressed six of the seven domains [25-27]. These latter two checklists excluded funding; the scale omitted data abstraction and had a "Partial" score for search strategy. The authors concluded that based on coverage of the seven domains that they considered key, these five systems (four checklists and one scale) represented "best practice" (i.e. were the best available instruments) for appraising systematic reviews. Although they considered other aspects of the systems, such as the methods used to select items and inter-rater reliability, they did not take these factors into consideration in their selection of these five systems, nor did they consider the suitability of the different systems for specific purposes.

The second review used a detailed process to evaluate and select a system and expanded the work by AHRQ up until the year 2005 [19]. They identified approximately 240 quality assessment instruments for systematic reviews, randomized controlled trials and observational studies as well as nearly 50 evidence grading systems. The instruments and systems identified were evaluated by type of study using the AHRQ evaluation grids from the first review, and considering descriptive items for most potential instruments and systems. The highest scoring instruments and systems from each grid represented the proposed selections. The proposed selections were then sent to the same experts that were contacted to review and provide comment during the initial expert consultation. Based on the second expert consultation, the AMSTAR 2005 was selected as the best instrument for appraising systematic reviews (Table 1). A description of the rationale for selecting that instrument is not available.

**Table 1:** A MeaSurement Tool to Assess Reviews (AMSTAR), 2005 (from COMPUS [19])

**1. Was an 'a priori' design provided?**
The research question and inclusion criteria should be established before the conduct of the review.
Yes
No
Can't answer
Not applicable
**2. Were there duplicate study selection and data extraction?**
There should be at least two independent data extractors and the consensus procedure for disagreements should be reported.
Yes
No
Can't answer
Not applicable
**3. Was a comprehensive literature search performed?**
At least two electronic sources should be searched. The report must include years and databases (e.g., Central, EPOC, and MEDLINE). Key words and/or MESH terms must be stated and where feasible the search strategy should be provided. All searches should be supplemented by consulting current contents, reviews, textbooks, specialized registers, or experts in the particular field of study, and by reviewing the references in the studies found.
Yes
No
Can't answer
Not applicable
**4. Was the status of publication (i.e., grey literature) used as an exclusion criterion?**
The authors should state that they searched for reports regardless of their publication type. The authors should state whether or not they excluded any reports (from the systematic review), based on their publication status.
Yes
No
Can't answer
Not applicable
**5. Was a list of studies (included and excluded) provided?**
A list of included and excluded studies should be provided.
Yes
No
Can't answer
Not applicable
**6. Were the characteristics of the included studies provided?**
In an aggregated form such as a table, data from the original studies should be provided on the participants, interventions and outcomes. The ranges of characteristics in all the studies analyzed (e.g., age, race, sex, relevant socioeconomic data, disease status, duration, severity, or other diseases) should be reported.
Yes
No
Can't answer
Not applicable
**7. Was the scientific quality of the included studies assessed and reported?**
'A priori' methods of assessment should be reported (e.g., for effectiveness studies if the author(s) chose to include only randomized, double-blind, placebo controlled studies, or allocation concealment as inclusion criteria); for other types of studies alternative items will be relevant.
Yes
No
Can't answer
Not applicable
**8. Was the scientific quality of the included studies used appropriately in formulating conclusions?**
The results of the methodological rigor and scientific quality should be considered in the analysis and the conclusions of the review, and explicitly stated in formulating recommendations.
Yes
No
Can't answer
Not applicable
**9. Were the methods used to combine the findings of studies appropriate?**
For the pooled results, a test should be done to ensure the studies were combinable, to assess the homogeneity (i.e., Chi-squared test for homogeneity, I^2^). If heterogeneity exists, random effects model should be used and/or the clinical appropriateness of combining should be taken into consideration (i.e., is it sensible to combine?).
Yes
No
Can't answer
Not applicable
**10. Was the likelihood of publication bias assessed?**
An assessment of publication bias should include a combination of graphical aids (e.g., funnel plot) and statistical tests (e.g., Egger regression test).
Yes
No
Can't answer
Not applicable
**11. Was the conflict of interest stated?**
Potential sources of support should be clearly acknowledged in both the systematic review and the included studies.
Yes
No
Can't answer
Not applicable

*Source*: AMSTAR 2005 (Beverley Shea, CIET, Institute of Population Health, Ottawa: personal communication, 2005 Oct)

### When and how should WHO undertake or commission new reviews?

There is wide agreement that guidelines should be informed by systematic reviews of the best available evidence among organisations that develop clinical practice guidelines and, increasingly, among organisations that develop guidance for population interventions (public health, health promotion, health systems and social interventions) [8,9,28-34]. Thus, priorities for systematic reviews are set, to some extent, when a decision is first made to develop recommendations. We reviewed the methodological literature relevant to priority setting for guidelines and health technology assessments, which overlaps largely with priority setting for systematic reviews, in our review on setting priorities for developing recommendations [4]. Additional questions related to undertaking or commissioning new reviews include: If there is a systematic review is it of good enough quality and recent enough that a new review is unlikely to be needed? Are there sufficient time and resources to commission or undertake a new review, if one is needed? If there is time, resources and a need for a new review, what is the best approach to getting the work done?

The first of these questions can be answered by considering the criteria discussed above and the likelihood of whether new research is likely to have been completed. Under some circumstances, it may not be warranted or possible to undertake or commission a systematic review even if there is not a previous systematic review; for example, for emerging diseases when it is known that the available evidence is sparse and when decisions must be made urgently.

We address which evidence should be used to address different types of questions in another paper in this series [35]. As we suggest in that paper, there is a cut-off point beyond which broadening the types of studies that are included requires a substantial investment of effort that will not yield additional information that usefully informs decisions. Similarly, there is a cut-off point beyond which more extensive searches are unlikely to yield additional useful studies.

An assessment of 159 systematic reviews with comprehensive literature searches found that the importance of trials that are difficult to locate may vary, but that generally in situations where resources are limited, thorough quality assessments should take precedence over extensive literature searches and translations of articles [36,37]. Consistent with this, another assessment of Cochrane reviews found that additional database searching beyond the Cochrane Central Register of Controlled Trials (CENTRAL) retrieved only a small percentage of extra trials, and that contacting authors and manufacturers to find unpublished trials appeared to be a more effective method of obtaining additional better quality trials [38].

Similarly, a third assessment of 20 Technology Assessment Reports by NICE found that a more selective approach to database searching would suffice in most cases and would save resources, whereas searching other sources, including contact with experts and checking reference lists, appeared to be a more productive way of identifying further studies [39]. Searching additional databases beyond the Cochrane Library, MEDLINE, EMBASE and SCI, plus BIOSIS limited to meeting abstracts only, was seldom found to be effective in retrieving additional studies for inclusion in the clinical and cost-effectiveness sections of Technology Assessment Reports (apart from reviews of cancer therapies, where a search of the ASCO database was recommended).

Information retrieval for systematic reviews for public health and other non-clinical interventions may be more elusive than retrieval for reviews in clinical medicine, due to the interdisciplinary nature of the research, use of research designs other than randomised trials, and limitations of what and how the research is indexed. While it may be important to consider other databases, strategies other than database searching are likely to be important [40,41]. Moreover, database searching in public health and other non-clinical areas may require specialised skills due to technical demands of the databases to be searched, lack of standardization of the vocabulary, and the relative scarcity of rigorous evaluations [42]. Information retrieval specialists may require a broad exposure to databases, the grey literature and the terminology that is used.

Several investigators have addressed the question of when a review or guideline needs updating [37,43-47]. French and colleagues found that of a sample of 254 updated Cochrane reviews 23 (9%) had a change in conclusion [43]. Another survey of Cochrane reviews found that of 104 updated reviews in the first half of 2003, 77% included no new data or data insufficient to influence the conclusion. In 16% new data had some impact on conclusions without major change, and in only 5% new data resulted in major changes in conclusions [44].

Johnston and colleagues, on the other hand, found that an updating strategy for cancer practice guidelines found 80 pieces of new evidence over a one-year period relating to 17 of 20 guidelines [45]. On average four pieces of new evidence were found per guideline, but there was considerable variation across the guidelines. Of the 80 pieces, 19 contributed to modifications of clinical recommendations in six practice guidelines, whereas the remaining evidence supported the original recommendations. In this case the updating process was resource intensive, but yielded important findings. However, it was possible to reduce the scope of the sources searched routinely to MEDLINE, the Cochrane Library and meeting proceedings. Another review of 17 guidelines published by AHRQ found that for seven guidelines new evidence and expert judgement indicated an update was needed, six were found to be in need of a minor update, three were considered still valid, and no conclusion was drawn for one [47]. The authors found that no more than 90% of the guidelines were still valid after 3.6 years and they estimated that about half the guidelines were outdated in 5.8 years. They concluded that guidelines should be reassessed every three years.

Comprehensive reviews are time-consuming. Many health technology assessment (HTA) agencies have established rapid assessment processes, particularly for new technologies [48-51]. There is no common definition of "rapid assessment" and there is variation in the scope, methods and time to complete assessments. While the concept is intuitively sound, there is little empirical evidence comparing alternative methods or comparing rapid assessments with more comprehensive methods. Milne and colleagues have described a range of HTA responses available in the UK, including 2–3 page assessments that take six weeks, rapid systematic reviews that take 8–10 weeks, technology assessment reviews that take six months, Cochrane reviews, and full HTA reports that take 3 years [52]. They identify three factors that determine the HTA response: what decision-makers want, including the time scale for decision making; the characteristics of the technology, including the importance of the uncertainty, the importance of the potential benefits, the rate of diffusion, and how much is already known from previous assessments; and the resources available for an assessment.

We did not find any evaluations of alternative methods for commissioning reviews or of comparisons between commissioning reviews and doing them in house. A survey of people preparing Cochrane reviews in Australia (with a response rate of 92/112) found that the most critical barriers to completion of a Cochrane review were lack of time (80%), lack of financial support (36%), methodological problems (23%) and problems with group dynamics (10%) [53].

### How should the findings of systematic reviews be summarised and presented to committees responsible for making recommendations?

The Conference on Guideline Standardization (COGS) developed an 18-item checklist for the reporting of guidelines [29]. The checklist includes the method for synthesizing evidence (how evidence was used to create recommendations, e.g., evidence tables, meta-analysis, decision analysis) and the recommendation grading criteria (the criteria used to rate the quality of evidence that supports the recommendations and the system for describing the strength of the recommendations).

The GRADE Working Group recommends the use of evidence profiles including detailed descriptions of the judgements used to assess the quality of evidence for each important outcome and a summary of the findings for each important outcome [54,55]. More recently the Cochrane Collaboration has developed summary of findings tables, based in part on GRADE evidence profiles [22,56].

All of these methods of presenting evidence to decision makers are based on consultations informed by evidence, such as comparisons of different ways of presenting evidence. We did not find comparisons of different ways of presenting evidence to groups developing recommendations.

In addition to summaries of the main findings, such as evidence profiles, the full systematic reviews should be available to both those making recommendations and to users of the recommendations [29]. These full systematic reviews should adhere to standards such as those recommended in the QUOROM statement [57].

### What additional information is needed to inform recommendations and how should this information be synthesised with information about effects and presented to committees?

Although there are a number of descriptive papers and guidelines for what additional information is needed in addition to systematic reviews of the effects of the options that are being considered, we did not find comparisons of alternative ways of synthesising this information and presenting it to groups making recommendations. As discussed in another article in this series [58], additional information that needs to be considered in a recommendation includes factors that might modify the expected effects, need (prevalence, baseline risk or status), values [59], costs and the availability of resources.

Methods of integrating this additional information and judgements include formal and informal consensus methods [60,61], decision analyses, and economic analyses [62,63]. Because factors such as modifying factors, needs and the availability of resources can vary greatly from setting to setting, methods for incorporating this information in global guidelines are particularly challenging. We did not find any evaluations of methods for addressing these challenges.

## Discussion

There is broad agreement on the need for systematic reviews to inform recommendations and on criteria for critically appraising systematic reviews. Several criteria have been identified that need to be considered when deciding whether a new systematic review is needed, including the needs of decision makers, the nature of the problem and the relevant interventions, and the availability of resources.

The available evidence suggests that, generally, in situations where time or resources are limited, thorough quality assessments should likely take precedence over extensive literature searches. When a full systematic review is not undertaken, for example because of the need for a rapid response, explicit consideration should be given to the need and urgency of undertaking a full systematic review and putting in place appropriate mechanisms for timely updating of the recommendations.

The frequency with which reviews or guidelines need to be updated is likely to vary, but as a rough rule of thumb, based in part on a study of clinical practice guidelines, the need for updating should be considered routinely after three years and more often for areas that are developing rapidly.

## Further work

Both the Agency for Healthcare Research and Quality and the Canadian Coordinating Office for Health Technology Assessment have funded projects on updating systematic reviews [64,65]. These reports should help to fill in some of the gaps in this review regarding when and how to undertake or commission an update of a review. Further work is needed on several of the other questions asked in this review, including evaluation of methods for rapid assessments, how best to present evidence to groups making recommendations and, importantly for WHO, how best to take into consideration information that varies from setting to setting when making global recommendations.

## Competing interests

ADO and AF work for the Norwegian Knowledge Centre forthe Health Services, an agency funded by the Norwegian government that produces systematic reviews and health technology assessments. All three authors are contributors to the Cochrane Collaboration. ADO and HJS are members of the GRADE Working Group. HJS is documents editor and chair of the documents development and implementation committee for the American Thoracic Society and senior editor of the American College of Chest Physicians' Antithrombotic and Thrombolytic Therapy Guidelines.

## Authors' contributions

ADO prepared the first draft of this review. HJS and AF contributed to drafting and revising it.
